# The Oslo Health Study: Is bone mineral density higher in affluent areas?

**DOI:** 10.1186/1475-9276-6-19

**Published:** 2007-11-23

**Authors:** Kari Alver, Anne J Søgaard, Jan A Falch, Haakon E Meyer

**Affiliations:** 1 Norwegian Institute of Public Health, Oslo, Norway; 2 Institute of General Practice and Community Medicine, Faculty of Medicine, University of Oslo, Norway; 3 Center of Endocrinology, Aker University Hospital, Oslo, Norway

## Abstract

**Background:**

Based on previously reported differences in fracture incidence in the socioeconomic less affluent Oslo East compared to the more privileged West, our aim was to study bone mineral density (BMD) in the same socioeconomic areas in Oslo. We also wanted to study whether possible associations were explained by socio-demographic factors, level of education or lifestyle factors.

**Methods:**

Distal forearm BMD was measured in random samples of the participants in The Oslo Health Study by single energy x-ray absorptiometry (SXA). 578 men and 702 women born in Norway in the age-groups 40/45, 60 and 75 years were included in the analyses. Socioeconomic regions, based on a social index dividing Oslo in two regions – East and West, were used.

**Results:**

Age-adjusted mean BMD in women living in the less affluent Eastern region was 0.405 g/cm^2 ^and significantly lower than in West where BMD was 0.419 g/cm^2^. Similarly, the odds ratio of low BMD (Z-score ≤ -1) was 1.87 (95% CI: 1.22–2.87) in women in Oslo East compared to West. The same tendency, although not statistically significant, was also present in men. Multivariate analysis adjusted for education, marital status, body mass index, physical inactivity, use of alcohol and smoking, and in women also use of post-menopausal hormone therapy and early onset of menopause, did hardly change the association. Additional adjustments for employment status, disability pension and physical activity at work for those below the age of retirement, gave similar results.

**Conclusion:**

We found differences in BMD in women between different socioeconomic regions in Oslo that correspond to previously found differences in fracture rates. The association in men was not statistically significant. The differences were not explained by socio-demographic factors, level of education or lifestyle factors.

## Background

Women and men in Oslo, the capital of Norway have the highest incidence rates of hip and forearm fractures and among the highest prevalence of vertebral fractures ever reported internationally [1-4]. In accordance with this, an European multi-centre study found that bone mineral density (BMD) in men and women in Oslo were among the lowest in Europe [5].

Within this high risk city of osteoporosis and osteoporotic fractures regional differences in hip fracture incidence have been reported [6]. The highest fracture rates are found in Eastern parts of the city, which is less affluent than the Western part of the city, with RR ranging from 1.23 to 1.67. Equivalent regional differences have been found regarding mortality and other health indicators [7]. These health inequalities are connected to differences between the regions regarding standard of living and socio-economic status of the habitants, with the highest annual income and highest level of education in West [8,9].

Socioeconomic status is well established as a determinant for several health aspects and disease outcomes. Regarding fracture risk, a study including white Americans 50 years and older, reported a linear decrease in hip fracture rates with increasing income after controlling for age and sex [10]. Likewise, an ecological study from Wales found higher fracture incidence among socio-economically deprived adults [11]. This effect diminished with age and was not observed in older age groups (above 75 years). In a Norwegian population based case-control study including men and women, low educational level was associated with increased risk of hip fracture [12]. This was not confirmed in a large Swedish case-control study among women [13]. They found however that being unemployed, being unmarried and living in an apartment (vs. one-family house) were associated with increased risk of hip fracture.

Studies addressing the association between BMD and socioeconomic status are likewise heterogeneous and not conclusive. Elliot et al found highest BMD in men from lower socio-economic groups [14], but several other studies have found an association of low BMD with low-income, low education and living in socially deprived areas [15-21]. These studies had limitations which could make it difficult to conclude and generalize – i.e. they included mainly postmenopausal women, they studied partly small samples and the participation was based on self-selection or referral from general practitioners/consultants.

Using data from a population-based health survey, we wanted to examine whether BMD was lower in the socioeconomic less affluent Oslo East compared to the more privileged Oslo West. Further, we wanted to assess whether the association could be attributed to socio-demographic factors, level of education or lifestyle factors.

## Methods

### Study population and data collection

All individuals living in the city of Oslo and born in 1970, 1960, 1955, 1940/41 and 1924/25 were invited to The Oslo Health Study (HUBRO) 2000–2001. Information about age, gender, address and marital status was obtained from the Population registry. A total of 8404 men (42.4%) and 10366 women (49.3%) participated [22]. HUBRO comprised a simple physical examination (measurements of blood pressure, pulse, height, weight, waist and hip circumference), collection of venous non-fasting blood samples and questionnaires. The questionnaires provided information on health status, symptoms, diseases and various aspects of health behaviour. Up to two reminders were sent to the non-responders of the invitation to participate in the survey.

### The osteoporosis sub-study

As part of HUBRO a random sample of the invited aged 30, 40, 45, 60 and 75 years was selected to a forearm BMD measurement by single x-ray absorptiometry. Of 1525 men aged 75 years 998 were reserved for follow-up of a previous population based survey [23], and thus not a part of this study. Of all those invited to bone densitometry 40% (1044 men and 1121 women) were measured. The attendance rate was lowest in the youngest age group and in men compared to women. Additional analyses comparing persons with a SXA densitometry with the rest of the HUBRO-population showed only minor differences regarding self perceived health, length of education, body mass index and physical activity. More detailed information on the attendance rate in the osteoporosis sub-study has been reported previously [24]. In the current paper we have excluded persons not born in Norway (487) to minimize interference on BMD from ethnicity. We have also excluded 368 persons born in 1970 (youngest age group), since most of them had not yet settled. In this age-group 83% had moved at least once during the last 5 years compared to 9–33% in the other age-groups. Three scans (1 man and 2 women) were not valid and information about place of residence was missing in 27 persons (7 men and 20 women). Finally 578 men and 702 women were included in the analysis.

### Bone Mineral Density assessment

BMD was measured by single energy x-ray absorptiometry (SXA; DTX-100, Osteometer MediTech Inc., Hawthorne, California). BMD was measured at both the distal and ultra-distal forearm site. In this study we have reported data at the distal site only (10–20% trabecular bone) to simplify the presentation since the results from the ultra-distal did not differ substantially. The distal site included a fixed length of 24 mm of both the radius and the ulna, starting distally at the point where the distance between ulna and radius is 8 mm. Bone densitometry was performed on the non-dominant forearm except in 1.8% of the cases where the non-dominant forearm was ineligible (cast, wounds). It has previously been shown that forearm BMD is only slightly higher at the dominant compared to the non-dominant side at both the distal and ultra-distal site, and thus no adjustments were done [25]. All scans were reviewed and reanalysed if necessary [26].

### Regions in Oslo

Initially we split Oslo into four regions – inner East, outer East, inner West and outer West – according to a previously used grouping [7]. The grouping was based on a social index taking the distribution of unemployment, education, non-Western immigrants and single parent into account. Thus Oslo was divided into four coherent regions where the population within the region had similar socio-economic status, living standard and house structure. Ranking the social status of the four regions from low to high we have in ascending order inner East, outer East, inner West and outer West. However, the major dividing line concerning health status and health related behaviour is between East and West [7]. We therefore decided on using two regions in our analyses – East and West (merging the two Eastern regions and the two Western regions) – because of the sample size and to simplify the presentation. Analyses based on four regions gave basically the same results.

### Risk factors

Marital status obtained from the invitation file was dichotomised into currently married vs. the others. Height and weight were measured at the screening station. Information about other risk factors was obtained by questionnaires. The participants were asked whether they were currently employed – and the answers were dichotomized into full time/part time vs. not employed. Total years of completed schooling/education were used as a continuous variable. The information about receiving disability pension (full/part) was dichotomized (yes or no). The question about weekly heavy physical activity (sweating and out of breath) in the spare time had four answer options, but was reduced to two – no activity vs. any activity. Frequency of alcohol consumption had eight answer categories which were reduced to four. Smoking status was dichotomized, current smokers vs. former and never smokers. Since the continuous variable "years since menopause" was highly correlated with age (r = 0.86), menopausal status was also dichotomized (before 50 years vs. 50 years and above) to avoid multicollinearity. Use of post-menopausal hormone therapy was categorised as current, former and never users. Information about present use of steroids (yes or no) was also obtained. In a sub-sample of the study population (359 women and 399 men 60 years and younger) we had information about physical activity at work, and in another sub-sample (506 women and 467 men) we had information about calcium intake from supplements and dairy products.

### Ethics and approvals

All the participants of the Oslo Health Study have given their written signed consent. The Norwegian Data Inspectorate has approved the Oslo Health Study, and it has been presented to the Regional Committee for Medical Research Ethics and conducted in full accordance with the World Medical Association Declaration of Helsinki.

### Statistics

All analyses were performed separately for men and women. Comparisons of age specific figures between socioeconomic regions were done by t-test for continuous data and by chi-square test for categorical data. The age-adjusted figures were compared using variance analysis. Standard deviations (SD) were reported for crude means. For age-adjusted means 95% confidence intervals were shown.

Mean BMD, prevalence of low BMD and prevalence of osteoporosis were compared between the socioeconomic regions. Low BMD was defined as having a BMD of one standard deviation or more under the age- and sex-specific means (Z score ≤ -1) in this population sample. Osteoporosis was defined as having a BMD of 2.5 standard deviations or more under the young female adult and young male adult means (T score ≤ -2.5). Participants aged 30–39 years in the Tromsø Health Study were used as a reference population [27]. Comparisons were done by chi-square tests. Logistic regression was employed to calculate the age-adjusted odds ratio of having low BMD and having osteoporosis.

Multiple regression analysis was used to study whether adjustment for other factors changed the association between BMD and socioeconomic region. The variables included in the regression models were age, marital status, level of education, employment status, received disability pension, body mass index, physical inactivity, use of alcohol and smoking. Use of post-menopausal hormone therapy and early onset of menopause were also included in the regression model for women. Because of missing data on some of the questions, 538 men and 626 women were included in the regression analysis.

## Results

A description of the sample is provided in table 1 and 2. In total, a larger proportion of men and women in Western parts of the city were married and a lower proportion was unemployed and received disability pension. Unemployment and disability pension were only analysed in those below the age of retirement, and the difference in unemployment between East and West was not statistically significant in women. Education was one of the criteria for grouping the districts into regions, and as expected, we found a substantial difference between the two regions in self-reported length of education in both genders – in favour of West. The inhabitants in the affluent Western part were somewhat taller than those living in Eastern parts of the city. In women, but not in men, body mass index was lower in West than in East. The prevalence of physical inactive persons and current smokers were higher in East than in West. Current use of post-menopausal hormone therapy was most common in 60 years old women, and more frequent in West compared to East.

**Table 1:** Characteristics of the study population – women. The osteoporosis sub-sample of the Oslo Health Study 2000–2001.

	**40/45 years**	**60 years**	**75 years**	**Total age-adjusted (CI)^b^**
**Women**				
Number of subjects				
West	126	93	80	299
East	179	91	133	403
Married (%)				
West	55*	63*	48	56 (50, 61)**
East	41	41	45	42 (38, 47)
Unemployed (%)				
West	13	23		17 (11, 22)
East	16	31		22 (17, 26)
Disability pension (%)				
West	4	20*		9.7 (5.1, 14.3)*
East	8	34		17.1 (13.0, 21.3)
Years of education^a^				
West	15.7 (3.2)*	13.3 (3.4)*	12.1 (3.1)*	13.9 (13.6, 14.3)***
East	13.5 (3.3)	11.0 (3.1)	9.3 (2.4)	11.6 (11.3, 11.9)
Height (cm)^a^				
West	168 (5.5)	166 (5.6)	161 (5.7)*	165.6 (164.9, 166.3)**
East	167 (5.7)	165 (5.8)	159 (5.9)	164.3 (163.7, 164.8)
Weight (kg)^a^				
West	66 (10.1)*	67 (9.2)	67 (14.3)	66.4 (65.1, 67.8)**
East	70 (12.7)	70 (12.5)	66 (11.3)	68.8 (67.7, 70.0)
Body mass index (kg/m^2^)^a^				
West	23.3 (3.2)*	24.6 (3.8)	25.7 (4.8)	24.4 (23.9, 24.9)***
East	25.3 (4.6)	25.8 (4.5)	26.2 (4.4)	25.7 (25.3, 26.1)
Physical inactive (%)				
West	15**	45	61	37 (31, 42)*
East	32	46	67	46 (41, 51)
Current smoker (%)				
West	26***	18	11	19 (14, 24)***
East	49	27	21	35 (31, 39)
Current use of HRT (%)				
West	6	47	13	21 (16, 25)*
East	7	40	8	14 (11, 18)

Oslo West is more privileged than Oslo East

*** p < 0.001, ** p < 0.005, *p < 0.05. Difference between West and East.

^a ^Figures are means (SD)

^b ^CI – 95% confidence interval

**Table 2:** Characteristics of the study population – men. The osteoporosis sub-sample of The Oslo Health Study 2000–2001.

	**40/45 years**	**60 years**	**75 years**	**Total age-adjusted (CI)^b^**
**Men**				
Number of subjects				
West	118	104	43	265
East	166	120	27	313
Married (%)				
West	60*	75*	74	67 (62, 73)*
East	48	63	70	56 (51, 61)
Unemployed (%)				
West	4*	16		10 (5, 14)*
East	12	20		16 (12, 20)
Disability pension (%)				
West	2*	9*		4.5 (0.5, 8.4)***
East	7	24		14.0 (10.5, 17.5)
Years of education^a^				
West	16.0 (3.2)*	15.2 (3.6)*	15.5 (3.0)*	15.7 (15.2, 16.1)***
East	13.5 (3.5)	11.5 (3.7)	11.9 (3.6)	12.6 (12.1, 12.9)
Height (cm)^a^				
West	183 (5.9)*	177 (6.1)	177 (6.2)	179.6 (178.8, 180.4)*
East	179 (6.7)	178 (7.1)	175 (10.0)	178.3 (177.6, 179.1)
Weight (kg)^a^				
West	85 (12.8)	82 (10.9)	81 (10.3)	83.5 (81.9, 85.0)
East	85 (13.8)	84 (13.0)	78 (12.1)	83.8 (82.4, 85.2)
Body mass index (kg/m^2^)^a^				
West	25.6 (3.4)	26.4 (3.2)	26.0 (3.3)	26.0 (25.6, 26.4)
East	26.4 (3.7)	26.7 (3.7)	25.8 (3.4)	26.5 (26.1, 26.9)
Physical inactive (%)				
West	14*	25*	33	20 (15, 26)**
East	26	38	46	33 (28, 38)
Current smoker (%)				
West	25*	17**	2**	19 (14, 24)***
East	40	37	26	37 (32, 42)

Oslo West is more privileged than Oslo East

*** p < 0.001, ** p < 0.005, *p < 0.05. Difference between West and East.

^a ^Figures are means (SD)

^b ^CI – 95% confidence interval

### Socioeconomic region

**Figure 1:**
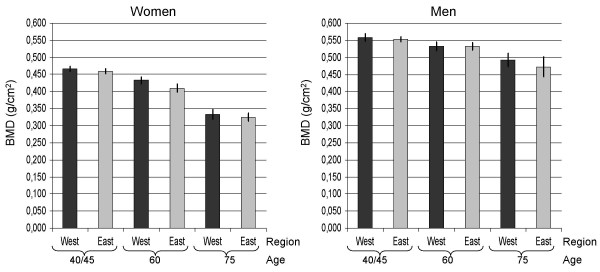
BMD in two sosioeconomic regions in Oslo. The Oslo Health Study.

In women, age adjusted mean BMD was lower in Oslo East than in Oslo West (table 3). The same pattern was present in all the three age groups, but the East-West difference was only statistically significant in women aged 60 years (figure 1). In men we observed the same tendency, but the differences were not statistically significant.

**Table 3:** Mean distal BMD, prevalence and odds ratio of low BMD^1 ^in Oslo West and Oslo East.

**Region**	**n**	**Mean BMD (95% CI)**	***Prevalence of low BMD (95% CI)***	***Odds ratio of low BMD (95% CI)***
**Women**				
West	299	0.419* (0.412 – 0.425)	11.7* (7.5 – 15.9)	ref
East	403	0.405 (0.400 – 0.410)	19.9 (16.3 – 23.5)	1.87 (1.22 – 2.87)*
**Men **				
West	265	0.540 (0.534 – 0.547)	13.0 (8.7 – 17.3)	ref
East	313	0.536 (0.530 – 0.542)	16.2 (12.2 – 20.1)	1.29 (0.81 – 2.07)

Oslo West is more privileged than Oslo East

The figures are age-adjusted.

^1 ^Low BMD is defined as z-score ≤ -1

* p < 0.05

The age-adjusted prevalence of low BMD (Z-score ≤ -1 SD) was higher in Oslo East than in Oslo West in both gender, but only statistically significant in women (table 3). The age-adjusted odds ratio of low BMD in women living in Oslo East compared with living in Oslo West was 1.87 (95% CI: 1.22–2.87). The age-adjusted odds ratio for having osteoporosis (T-score ≤ -2.5 SD) was 1.12 (95% CI: 0.63–1.99) in men and 1.55 (95% CI: 0.94–2.54) in women. In women aged 60 the corresponding odds ratio for osteoporosis was 3.70 (95% CI: 1.16–11.84) (osteoporosis figures are not shown in table).

Adjusting for age and BMI in multiple regression analysis slightly increased the difference in mean BMD between Oslo East and Oslo West in women (table 4). Further adjustment for other known risk factors for BMD gave almost the same result as the solely age-adjusted figures. Apart from socioeconomic region, age (β = -0.004, p < 0.001), body mass index (β = 0.003, p < 0.001) and current use of estrogens (β = 0.028, p < 0.001) were associated with BMD in women. Adding employment and disability pension to the full model, thus excluding the oldest age-group, gave a statistically significant difference in mean BMD between East and West of 0.014 g/cm^2 ^(data not shown in table). Including physical activity at work for those 60 years and younger did not change the result notably.

**Table 4:** Differences in BMD between Oslo West and Oslo East adjusted for age and other confounders.

	***BMD^1 ^(95% CI)***	***BMD^2 ^(95% CI)***	***BMD^3 ^(95% CI)***
**Women **(n = 626)	0.013 (0.004 – 0.021)	0.016(0.007 – 0.024)	0.012(0.003 – 0.021)
**Men **(n = 538)	0.004 (-0.006 – 0.014)	0.004 (-0.005 – 0.014)	0.003(-0.008 – 0.014)

Oslo West is more privileged than Oslo East

^1^Age-adjusted. This figure differ from that in table 3 because of uneven numbers included in the analysis

^2^Adjusted for age and body mass index.

^3^Adjusted for age, marital status, level of education, body mass index, physical inactivity, smoking status and use of alcohol. For women the model was also adjusted for use of hormone replacement therapy (current and previous) and early onset of menopause.

In men we found no socioeconomic regional differences in BMD after controlling for marital status, level of education, body mass index, physical inactivity, use of alcohol and smoking in addition to age. Apart from age (β = -0.002, p < 0.001), being physically active (β = 0.012, p = 0.03) were the only covariate significantly associated with BMD in men. When adding employment status, disability pension and physical activity at work to the full model in men 60 years and younger, there was still no significant difference between East and West.

In a sub-sample we had information about calcium intake through dairy products and supplements [28]. Adding calcium in the regression model did not change the effect of socioeconomic region on BMD in either gender – neither did controlling for use of steroids.

## Discussion

We found lower BMD in women in the socioeconomic less affluent Oslo East compared to the more privileged Oslo West. In the age specific analysis the difference was only significant in women aged 60 years. In men the difference was less pronounced and not statistically significant. The difference in BMD in women between the socioeconomic regions persisted after controlling for known risk factors. Individual differences in socio-demographic and lifestyle factors did not explain the regional differences we found.

Socioeconomic region assign all persons living in the same geographical area to the same socioeconomic position independent of individual socioeconomic characteristics. Since the effect of socioeconomic region on BMD in women sustain after adjusting for years of education and other covariates, there are probably other factors associated or influenced by socioeconomic region affecting BMD than those we have included in our model. Nutritional and neighbourhood factors are not controlled for, and common environmental factors (e.g. lead exposure) and water supply source are unexplored possible explanations.

From an individual perspective the socioeconomic differences we found in BMD do not seem substantial – at least not compared to other health outcomes and risk factors such as cardiovascular disease and smoking. However, small differences in BMD between regions may have large impact on fracture incidence in the population. A difference in BMD of e.g. 0.014 g/cm^2 ^in women, which is the difference in age-adjusted mean BMD between East and West, amount to about 1/4 SD. In a meta-analysis Marshall et. al showed that the relative risk for hip fracture in women was 1.8 per SD decrease in distal forearm BMD [29]. Roughly estimated a decrease of 1/4 SD in BMD may increase the risk of hip fracture by about 20%. In women it seems that the difference in BMD in our study concurs with the risk estimates from the hip fracture study in Oslo – 23% increased risk of hip fracture in women living in inner East compared with inner West [6]. In men the corresponding fracture difference was 67%, which might suggest that other factors than BMD is of importance in explaining fracture risk differences between socioeconomic regions. However, these comparisons should be interpreted with caution since we cannot fully compare the regions in our study with those in the hip fracture study in Oslo.

Few published studies have focused on socioeconomic differences in BMD and some of them have obvious limitations as for example over-representation of well-educated individuals [19,20] or comparison of groups of unequal ethnic composition [15]. An American study reported no association between BMD in the forearm and education in women above 65 years [30]. In another American study they found a positive education-BMD association in black, but not in white and Hispanic postmenopausal women. However, they found an income-BMD association in white, but after adjustments for behavioural factors, associations with education and income were eliminated [21]. Our results corresponds, however, to other studies which reported a relation between educational level and BMD in postmenopausal women [16,17,20], although a Chinese study reported education-BMD association in weight bearing sites only and not in the arms [17].

Socioeconomic differences in BMD in men are particularly scarcely explored, possibly reflecting their lower incidence of osteoporotic fractures. Elliot et al found highest BMD in men from lower socio-economic groups [14]. In that study a larger percentage of those in the lower socioeconomic groups were employed in manual labour, but that did not explain the differences they found. A negative association between education and hip BMD in old men was also found in an American study, but the effect of education was eliminated after adjustment for weight [31].

It should be noted that the socioeconomic distribution of risk factors for BMD might differ between different populations. In the Italian study smoking were more common in those with high educational level [20] and in the Turkish study those in the lower socioeconomic group had lower alcohol intake and smoked less than the others [18]. The distribution of risk factors between socioeconomic groups may contribute to blur the effect of socioeconomic position on BMD. A risk factor for many health conditions such as high body mass index, which is more prevalent in lower socioeconomic groups, is a protective factor for BMD. In our study, neither body mass index nor any of the other confounding factors added in the full multivariate model could explain the difference in BMD between the socioeconomic regions (table 4). Neither could educational level, which was one of the criteria for constructing the two regions. Further research is needed to investigate other factors influencing people's bone health in the different socioeconomic regions in Oslo.

The strength of this study is the population-based setting and the bone measurements in both men and women in the age-range 40–75 years. In addition we had information on the most important socio-demographic and lifestyle factors that is known to be associated with BMD. Further, the grouping of socioeconomic region was based on an index of social- and health-related indicators, which has previously been used to show large differences in mortality between Oslo West and Oslo East [7].

On the other hand, although the overall population size was large enough to detect differences in BMD of about 0.015 g/cm^2 ^with power 80% and 5% significance level, we did not have enough power to detect small differences in the age groups with the fewest participants (i.e. men 75 years). Furthermore, the attendance rate was generally low in both the main survey and the BMD sub-study and selection bias cannot be excluded. Attendance was positively associated with age, income, education, living in Outer West and Outer East. However, the impact of self-selection in the Oslo Health Study has been evaluated and the prevalence estimates of factors associated with BMD such as body mass index, smoking and self perceived health was found to be quite robust [22]. The associations between disability pension (as a proxy of unhealthy persons) and several background variables, including level of education and region in Oslo, were compared in attendees and non-attendees and were found to be unbiased in Norwegian born individuals [22].

Another limitation of our study was that BMD was measured at the forearm. The NORA study has, however, shown that peripheral measurements, including the forearm, have a strong relationship to later osteoporotic fracture [32]. Forearm SXA is also one of the most precise bone densitometric methods [33].

## Conclusion

In conclusion, we found socioeconomic differences in BMD in women, but not in men. The differences between socioeconomic regions in women were not explained by education, lifestyle or the other recorded covariates.

## Competing interests

The author(s) declare that they have no competing interests.

## Authors' contributions

KA carried out the data analyses and wrote the manuscript. AJS was the project manager of the Oslo Health Study. She also revised the manuscript and assisted in the data analyses. JAF contributed to interpretation of the data and revised the manuscript. HEM was responsible for the Osteoporosis sub study and contributed to the data analyses and writing process. All authors were involved in planning of the study, in the discussion of the methods and results, and all have read and approved the final manuscript.
